# Follow‐up and growing‐up formula promotion among Mexican pregnant women and mothers of children under 18 months old

**DOI:** 10.1111/mcn.13337

**Published:** 2022-03-15

**Authors:** Mireya Vilar‐Compte, Sonia Hernández Cordero, Ana C. Castañeda‐Márquez, Nigel Rollins, Gillian Kingston, Rafael Pérez‐Escamilla

**Affiliations:** ^1^ Department of Public Health Montclair State University Montclair New Jersey USA; ^2^ Research Institute for Equitable Development (EQUIDE) Universidad Iberoamericana Mexico City Mexico; ^3^ School of Public Health of Mexico National Institute of Public Health Cuernavaca México; ^4^ Department of Maternal, Newborn, Child and Adolescent Health Geneva Switzerland; ^5^ Executive Fellow, School of Business Kings College London UK; ^6^ Department of Social and Behavioral Sciences Yale School of Public Health New Haven Connecticut USA

**Keywords:** breastmilk substitutes, follow‐up formula, growing‐up milks, infant formula, marketing, Mexico, promotion, theory of reasoned action

## Abstract

Milk formula sales have grown globally, particularly through follow‐up formulas (FUF) and growing‐up milks (GUM). Marketing strategies and weak regulatory and institutional arrangements are important contributors to caregivers' decisions about child feeding choices. This study describes maternal awareness, beliefs, and normative referents of FUFs and GUMs among Mexican pregnant women and mothers of children 0–18 months (*n* = 1044) through the lens of the theory of reasoned action (TRA). A cross‐sectional survey was undertaken in two large metropolitan areas of Mexico. Descriptive analyses were conducted following the constructs of the TRA. One‐third of the participants had heard about FUFs, mainly through health professionals (51.1%) and family (22.2%). Once they had heard about FUFs, the majority (80%) believed older infants needed this product due to its benefits (hunger satisfaction, brain development, and allergy management). One quarter of the participants were already using or intended to use FUFs; the majority had received this recommendation from doctors (74.6%) and mothers/mothers‐in‐law (25%). Similarly, 19% of the women had heard about GUMs. The pattern for the rest of TRA constructs for GUMs was similar to FUFs. Mexican women are exposed to FUFs and GUMs, once women know about them, the majority believe older infant and young children need these products, stating perceived benefits that match the poorly substantiated marketing claims of breast‐milk substitutes. Health professionals, particularly doctors, act as marketing channels for FUFs and GUMs. Marketing of FUFs and GUMs represents a threat to breastfeeding in Mexico and a more protective regulatory and institutional environment is needed.

## Key messages

- Mexican pregnant women and mothers are highly exposed to FUFs and GUMs.
- Most women who know FUFs and GUMs believe older infants and young children need these products.
- The perceived benefits of FUFs and GUMs match those displayed in BMS marketing.
- Health providers are being used as marketing channels for FUFs and GUMs.
- Stronger regulatory and institutional frameworks for BMS are urgently needed in Mexico.

## Graphical abstract

Behavioral framework of the intention to use/use of follow‐up formulas and growing‐up milks among Mexican pregnant women and mothers of infants and young children.

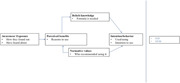

## 1 INTRODUCTION

Commercial milk for infants and young children refers to milks intended for children younger than 2 years of age, which is the minimum recommended breastfeeding period. Some of these products are sometimes labelled as suitable for older children (Pereira et al., 2016). Commercial milk market includes three common products that are marketed as replacements for breast‐milk (WHO, 1981): infant formulas (IF), defined as breast‐milk substitutes designed to fulfil the requirements of an infant during the first months of life (Codex Alimentarius, 1991); follow‐up formula (FUF), a liquid prepared from the milk of cows or other animals, and/or other constituents of animal, and/or plant origin proteins, intended to be part of the weaning diet of young children 6–12 months old; and growing‐up or toddler milk (GUM), that are similar products intended for children 1–3 years old and usually expected to replace breast‐milk and cow's milk (Hernández‐Cordero et al., 2019).

Based on Euromonitor data, prior studies have estimated that milk formula sales grew globally by 121.5% between 2005 and 2019, from 3.5 to 7.4 kg/child (Baker et al., 2020). This market growth has been largely attributed to GUMs' sales, which grew by 220% over the same period (Pries et al., 2021). The market growth of formulas has shown regional variations, being largest in East Asia and the Pacific, both for FUFs and GUMs; followed by Middle East/North Africa and South Asia for FUFs; and Eastern Europe, and the Middle East/North Africa for GUMs (Baker et al., 2020; Coriolis Research, 2014). There is increased concern about the growth in sales of these products, especially GUMs, since, although described by the manufacturers as advantageous, these products are not necessary and not recommended at any age (McCann et al., 2020). Prior studies document that GUMs (i.e., toddler milks) consist primarily of powdered milk, vegetable oil, and corn syrup solids or other caloric sweeteners (Harris et al., 2017). The added sugars in these products are not recommended for children (WHO, 2017). Furthermore, a recent analysis has found that these products have higher energy, carbohydrate and total sugar levels than full‐fat cow's milk (McCann et al., 2020). In fact, GUMs have been reported to be comparable to soft drinks when it comes to total sugar content (Bridge et al., 2020). However, the marketing strategies behind these products position them as an optimal nutrition solution to caregivers (Harris & Pomeranz, 2020).

Prior evidence shows that FUF and GUM advertisements use strategies such as line extension (introducing additional items in a given product category under the same brand name; Armstrong et al., 2014) and cross‐promotion (manufacturer's attempts to sell the consumer new or other products related to a product the consumer already uses; WHO, 2010) between IF, FUF, and GUM to portray “formulas” as a common product, affecting mothers' perceptions about choices of infant and young child feeding methods. This has been regarded as a deliberate strategy to evade restrictions on advertising. More specifically, in many countries, GUMs can be legally advertised, and this can be misunderstood by pregnant women and mothers as also promoting infant formula (Cattaneo et al., 2015). In 2016, the World Health Assembly passed Resolution 69.9, the associated WHO Guidance on ending the inappropriate promotion of foods for infants and young children, which included GUMs in its definition of breast‐milk substitutes (BMS), therefore falling under the International Code of Marketing of Breast‐milk Substitutes (hereafter, The Code). However, only 16% of countries that regulate BMS marketing explicitly ban marketing of GUMs (Choi et al., 2020; WHO, 2018).

Cross‐promotion is of concern because promotion of IF, FUF and GUM manufactured by the same companies (i.e., branding) can lead caregivers to mistakenly provide GUMs to infants, which do not meet their nutritional requirements (Romo‐Palafox et al., 2020). In addition, cross‐promotion can also foster the perception that IF, FUF and GUM are products that are needed beyond the first year of life (Pereira et al., 2016; Romo‐Palafox et al., 2020).

Although regulations intend to protect the health of children, there is evidence that they are inadequate and are not working to prevent such unethical BMS marketing (Ching et al., 2021; Hernández‐Cordero et al., 2019). Prior studies have suggested the need of renewed efforts towards implementation, monitoring, and enforcement of The Code and subsequent World Health Assembly resolutions (Baker et al., 2020), as well as more transparent labelling (Pereira et al., 2016). In terms of labelling, a recent global study analysing labelling of BMS reported that labelling is unclear and inconsistent across brands and between countries, and concludes that BMS need to have a mandatory regulation of sugar content with clear front‐of‐package (FOP) nutrition information (Bridge et al., 2020). This could help caregivers make better‐informed choices about infant and young child feeding. Such information is also fundamental, as the costs of FUF and GUM can take a substantial share of families' expenditure, especially in low‐ and middle‐income countries, which can potentially affect access to other healthful choices and contribute to food insecurity in the household.

However, given the outlook of increases in FUFs and GUMs' sales globally, it is fundamental to better understand how the intentions to use or actual use of these products is structured and influenced by marketing. Hence, the aim of the current research is to describe maternal awareness, beliefs and normative referents of FUFs and GUMs among Mexican pregnant women and mothers of children under 18 months old through the lens of the theory of reasoned action (TRA) (Swanson & Power, 2005). More specifically, we seek to inform the level of awareness about the existence of these products among pregnant women and mothers; how women find out about FUFs and GUMs; their beliefs about the need of them as part of their child's nutrition; their intention or actual behaviour of use; the benefits perceived in using FUFs and GUMs; and the referent subjects shaping their beliefs and use of these products. Findings from this study are expected to inform policies designed to further protect, promote, and support breastfeeding in Mexico, a country where most women choose to breastfeed their infants but many of them cannot do it for as long as recommended.

## 2 MATERIAL AND METHODS

### 2.1 Study design and research setting

We performed a descriptive study based on a cross‐sectional survey of mothers 18 years and older who were pregnant (3 months or more) or who had children 0–18 months old. The survey focused on children aged 0–18 months to ensure that there were enough feeding modalities throughout key stages of infant and young children nutrition. Data was collected through nonprobabilistic sampling in two large metropolitan areas of Mexico, Mexico City and Guadalajara. These cities were selected due to their population size and diversity in terms of socioeconomic characteristics, ethnicity, and cultural aspects. Within each of the cities, study areas (municipalities and boroughs) were selected based on their aggregate socioeconomic status (i.e., low, medium, or high socioeconomic level). A total of 1050 women participated, *n* = 525 in each city. Women were recruited based on predefined quotas targeted at achieving a diverse sample with regard to: pre‐ and postnatal status, infant age, household socioeconomic level, and city of residence. Recruitment took place at hospitals, both public and private, as well as in child daycares, pharmacies and stores that sell baby products.

Data on infant and young child feeding practices were collected through a previously validated questionnaire which included information about sociodemographic characteristics and infant and young child feeding intentions and practices. Questionnaires were applied face‐to‐face by trained interviewers between January and May 2020. The questionnaire was piloted to assess its understanding and feasibility, and to verify if interviewers were ready to start applying it. Before applying the questionnaire, participants signed an informed consent that explained the purpose of the project. The protocol was approved by the ethics committee of the World Health Organization (WHO) and by the Research Ethics Committee of the National Institute of Public Health in Mexico.

The analytical sample for the current analyses was *n* = 1044; six cases were excluded because these mothers had both an infant under 18 months old and were also pregnant at the time of the interview.

### 2.2 Theoretical model

**Figure 1:**
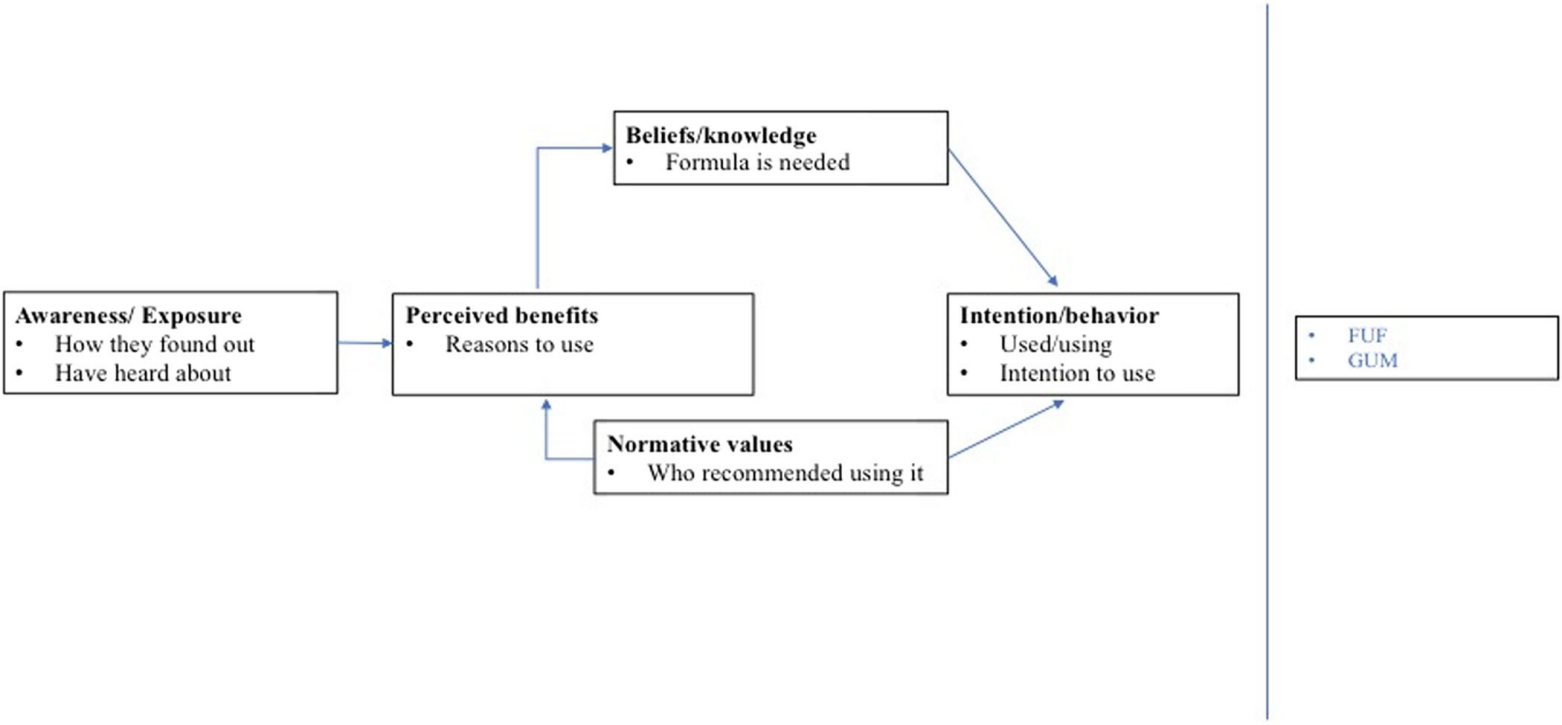
Behavioural framework about intention to use/use of FUFs and GUMs among Mexican women. FUF, follow‐up formula; GUM, growing‐up milk

We used the TRA model to guide the analytical strategy (Figure 1), which strongly focuses on intention as a key determinant of behaviour. Also, the TRA model posits that behavioural intention is determined by *attitudes* toward performing behaviour (i.e., beliefs about outcomes/attributes of performing the behaviour) and *subjective norms* associated with a behaviour, that are highly influenced by referent individuals (i.e., relevant individuals who approve or disapprove a behaviour such as friends, family, doctors, coaches, etc.). The TRA was useful for our purposes because this is a descriptive analysis without assumptions of volitional control over the behaviour of interest.

### 2.3 Measures

**Figure 2:**
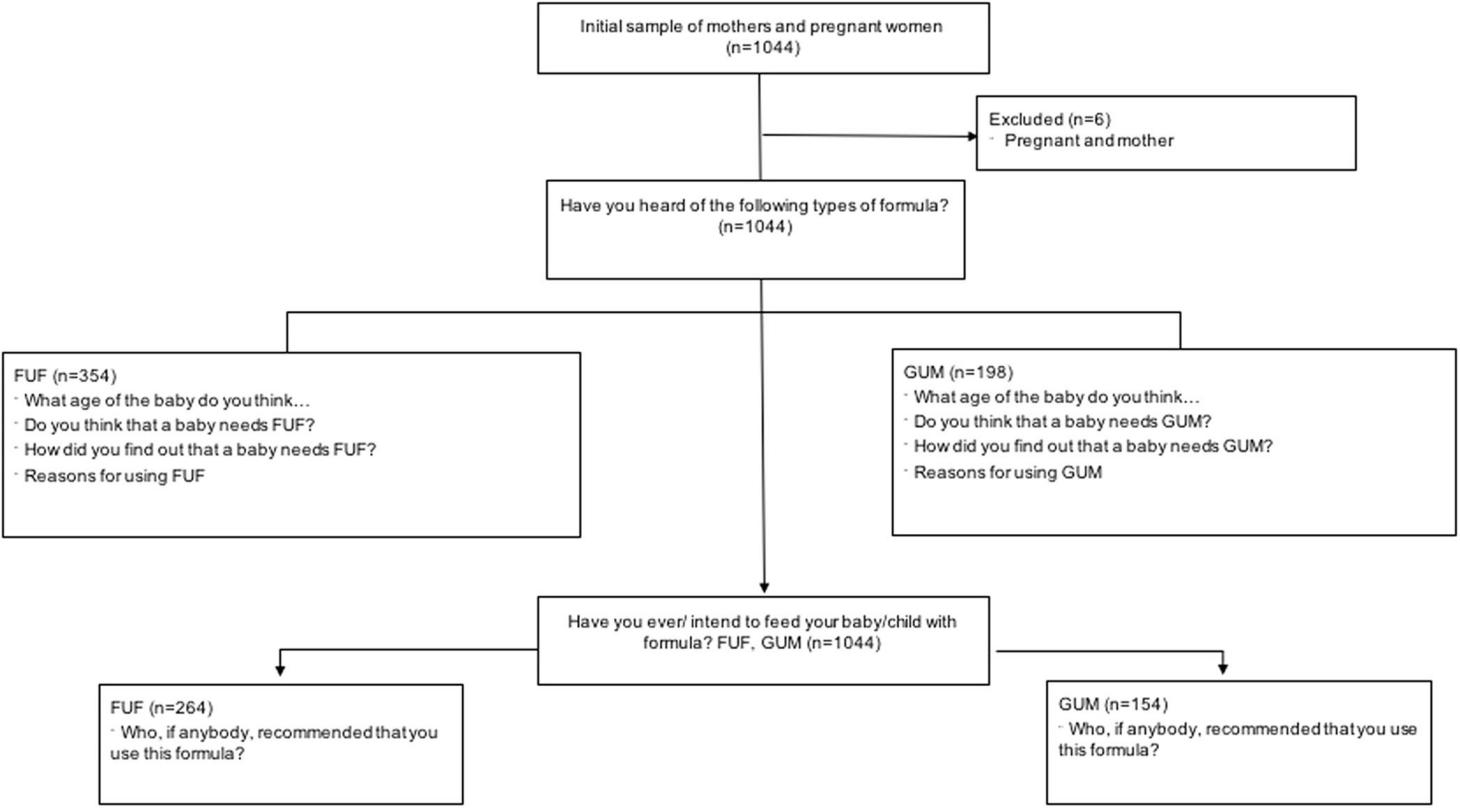
Samples' size flow chart. Follow‐up and growing‐up formula promotion among Mexican pregnant women and mothers of children under 18 months

Our analysis is based on the following groups of variables (Figure 2): (i) To assess environmental factors, women were asked if they had heard about FUFs or GUMs, which can be understood as a proxy measure of conscious exposure. This variable was operationalized as women answering yes, no, or don't know. (ii) Women who stated that they had heard about FUFs or GUMs were asked about how they found out about these products. For analytical purposes, we classified this variable into the following categories: friends and other mothers; family members; health providers; social media, helplines or baby clubs; advertisements; other; and don't know. (iii) Furthermore, to assess the behavioural belief domain of the TRA model, women who reported having heard about FUFs and GUMs, were also asked whether they believed that older infants and young children needed FUF and GUM, and this was operationalized as answering yes, no, or don't know. (iv) To assess behavioural outcomes, women who had heard of FUFs and GUMs were asked about the perceived benefits of these products. To answer this question, they could ascertain more than one perceived benefit, including infant behaviours, such as avoiding the infant/young child feeling hungry, crying less, or sleeping better; common marketing nutritional benefits, such as the perceptions that FUFs and GUMs providing more iron, prebiotics, and DHA; benefits for the infant and young child's development, such as perceived contributions of FUFs and GUMs to brain development, baby's future potential, and growth; perceived benefits linked to allergy management; and perceptions about breast‐milk not being good enough or cow's milk having less nutrients than BMS. In addition, they could choose to respond with the ‘other benefits’ or ‘don't know’ options. (v) All women who participated in the study were asked about their intention to use or if they currently used FUFs and GUMs. (vi) Finally, women who stated intentions to use or actual use of FUFs and GUMs were asked about who had recommended these products. From a behavioural theory perspective, this is a proxy of a normative referent. Women could identify more than one source, including spouse, mothers or mothers‐in‐law, other family members, friends, social media, people in their community, through people they follow on TV, radio or social media, science experts, doctors, nurses or midwifes, and pharmacists.

### 2.4 Analysis

Analyses were performed separately for FUFs and GUMs. In both cases, descriptive analyses and proportions stratified by gravidity (i.e., pregnant or mother of children under 18 months old) were estimated, and differences were assessed through *t*‐test, *χ* ^2^ and Fischer exact tests. Analyses were also stratified by parity, but as outcomes were very similar as those stratified by gravidity, these are not presented. All analyses were conducted in STATA 15 (StataCorp., 2017).

## 3 RESULTS

The study sample comprised 1044 women, of whom 296 were pregnant and 748 had a child between 0 and 18 months old at the time of the study. The sociodemographic characteristics were similar in both groups (Table 1). About half of the mothers had secondary school or less, 37% had completed high school, and only 12% had some graduate level education. Approximately 30% of the participants reported being employed at the time of the interview, and this percentage was only 2% lower among pregnant women. As expected per study design, about one‐third of the women in each group were in the low, medium and high socioeconomic groups. On average, pregnant women were significantly younger than mothers by about 1.5 years, and a significantly larger number of pregnant women were primiparas (67.2% compared to 43.0% among mothers of children 0‐18 months old).

**Table 1:** Sociodemographic characteristics of the study sample stratified by pregnancy/motherhood

	Total (*n* = 1044)	Pregnant women (*n* = 296)	Mothers w/children 0–18 m (*n* = 748)	*p* value
Mother's age, mean (SD)	27.23 (6.38)	26.15 (6.25)	27.66 (6.39)	<0.001
Education level, *n* (%)				
Secondary or less	525 (50.29)	151 (51.01)	374 (50.00)	0.98
High school	393 (37.64)	109 (36.82)	284 (37.97)	
Graduate or more	126 (12.07)	36 (12.16)	90 (12.03)	
Employment, *n* (%)				
Yes	514 (49.23)	85 (28.72)	229 (30.61)	0.54
No	730 (69.92)	211 (71.28)	519 (69.39)	
Socioeconomic status, *n* (%)				
Low	348 (33.33)	99 (33.45)	249 (33.29)	0.99
Middle	349 (33.43)	99 (33.45)	250 (33.42)	
High	347 (33.24)	98 (33.11)	249 (33.29)	
Parity, *n* (%)				
Primiparous	521 (49.90)	199 (67.23)	322 (43.05)	<0.0001
Multiparous	523 (50.10)	97 (32.77)	426 (56.95)	

Overall, one‐third of the participants recalled having heard about FUFs; this proportion was significantly larger among mothers with children under 18 months (38.9%), compared to pregnant women (21.8%) (Table 2). Women had heard about FUFs mainly through health professionals (51.1%), family (10.5%), and friends or other mothers (8.2%). Due to how the survey was structured, it was not feasible to describe more specifically what type of health providers mothers have heard about FUFs from, as this was a generic category. These proportions were significantly different between mothers and pregnant women. Mothers were more likely than pregnant women to find out about FUFs through health professionals (57.0% vs. 23.81%). By contrast, pregnant women were more likely to hear about FUFs from family and friends or other mothers, than mothers (22.2% and 14.3%, respectively, compared to 6.9% and 7.9% among mothers). Once a woman heard about FUFs, the great majority (approximately 80%) believed that older infants needed this product. There were no significant differences in this belief between pregnant women and mothers. The most frequently reported benefits of FUFs were: (i) that they helped older infants be less hungry (24.9%), (ii) that they were good for brain development and future potential (16.9% and 26.8%, respectively), and (iii) that they benefited allergy's management (20.62%). The perceptions of these benefits were not statistically different between pregnant women and mothers.

**Table 2:** Descriptive behaviour characteristics around follow‐on formulas (FUF) among Mexican women, stratified by pregnancy/motherhood status

	Total	Pregnant women	Mothers w/children 0–18 m	p value
Sample	*n* = 1044	*n* = 296	*n* = 748	
Having heard about FUF, *n* (%)				
Yes	354 (33.91)	63 (21.8)	291 (38.90)	<0.0001
No	689 (66.00)	233 (78.72)	456 (60.96)	
Don't know	1 (0.10)	0 (0.00)	1 (0.13)	
Intention or actual use of FUF, *n* (%)				
Yes	264 (25.29)	38 (12.84)	226 (30.21)	<0.0001
No	780 (74.71)	258 (87.16)	522 (69.79)	
*Among those who have heard of FUF*				
Sample	*n* = 354	*n* = 63	*n* = 291	
How did you find out about FUF, *n* (%)				
Friends & other mothers	29 (8.19)	9 (14.29)	20 (6.87)	0.05
Family	37 (10.45)	14 (22.22)	23 (7.90)	0.001
Health professionals	181 (51.13)	15 (23.81)	166 (57.04)	<0.0001
Social media, helpline, baby club	11 (3.11)	1 (1.59)	10 (3.44)	0.69
Advertisements	25 (7.06)	7 (11.11)	18 (6.19)	0.17
Other	50 (14.12)	13 (20.63)	37 (12.71)	0.10
Don't know	21 (5.93)	4 (6.35)	17 (5.84)	0.77
Belief baby needs FUF, *n* (%)				
Yes	287 (81.07)	51 (80.95)	236 (81.10)	0.11
No	64 (18.08)	10 (15.87)	54 (18.56)	
Don't know	3 (0.85)	2 (3.17)	1 (0.34)	
Perceived benefits of FUF, *n* (%)				
Perceived baby's behaviour				
Less hungry	88 (24.86)	12 (19.05)	76 (26.12)	0.23
Less crying	10 (2.82)	2 (3.17)	8 (2.75)	0.69
Better sleep	27 (7.63)	5 (7.74)	22 (7.56)	0.99
Perceived nutritional benefits				
Iron	45 (12.71)	8 (12.70)	27 (12.71)	0.99
Prebiotics	7 (1.98)	2 (3.17)	5 (1.72)	0.61
DHA	11 (3.11)	1 (1.59)	10 (3.44)	0.69
Perceived benefits for baby's development				
Brain development	60 (16.95)	8 (12.70)	52 (17.87)	0.32
Baby's future	95 (26.84)	16 (25.40)	79 (27.15)	0.77
Baby's growth	48 (13.56)	11 (17.46)	37 (12.71)	0.31
Perceived need due to…				
Allergies	73 (20.62)	17 (26.98)	56 (19.24)	0.16
Breast‐milk not enough benefits	17 (4.80)	3 (4.76)	14 (4.81)	0.99
Cow's milk not enough nutrients	24 (6.78)	4 (6.35)	20 (6.87)	0.99
Other perceived benefits	15 (4.24)	2 (3.17)	13 (4.47)	0.99
Don't know	35 (9.89)	5 (7.94)	20 (10.31)	0.56
*Among those intending or using FUF*				
Sample	*n* = 264	*n* = 38	*n* = 226	
Who recommended using FUF, *n* (%)				
Spouse	8 (3.03)	1 (2.63)	7 (3.10)	0.99
Mother/Mother‐in‐law	66 (25.00)	15 (39.47)	51 (22.57)	0.02
Other family members	33 (12.50)	9 (23.68)	24 (10.62)	0.03
Friends	25 (9.47)	9 (23.68)	16 (7.08)	0.004
Social media	2 (0.76)	0 (0.00)	2 (0.88)	0.99
People in the community	3 (1.14)	1 (2.63)	2 (0.88)	0.34
People I follow on TV/Radio/Social Media	4 (1.52)	1 (2.63)	3 (1.33)	0.46
Science experts	4 (1.52)	3 (7.89)	1 (0.44)	0.01
Doctors	197 (74.62)	19 (50.00)	178 (78.76)	<0.0001
Nurses/Midwifes	4 (1.52)	0 (0.00)	4 (1.77)	0.99
Pharmacists	4 (1.52)	1 (2.63)	3 (1.33)	0.46

One quarter of women from the full sample stated their intentions to use or their actual use of FUFs (Table 2); this proportion was significantly higher among mothers than pregnant women (30.2% and 12.8%, respectively). Information about who recommended the product (FUFs) allowed dissaggregation by type of health providers (i.e., nurses, doctors, pharmacists), as well as other nonmedical sources. Among women intending or using FUFs, it was recommended by doctors (74.6%), mothers and mothers‐in‐law (25%), and other family members (12.5%). Pregnant women were significantly more likely than mothers to have been influenced by mothers and mothers‐in‐law (39.5% vs. 22.5%), other family members (23.7% vs. 10.6%), and friends (23.6% vs. 7.1%). On the other hand, among mothers, doctors were significantly more likely to have had recommended FUFs than among pregnant women (78.8% vs. 50%, respectively).

For GUMs (Table 3), less women reported having heard of these products when compared to FUFs. However, 19% of the participants reported having heard of GUMs, and this proportion was significantly higher among mothers than pregnant women (21.4% vs. 12.8%). Similar to FUFs, women reported that they had heard about GUMs mainly through health providers (43.4%) and family (15.7%), although advertisements were also a relevant source of exposure (9.6%). As in the case of FUFs, a larger proportion of mothers had heard about GUMs through health providers when compared to pregnant women (49.4% vs. 18.4%). However, the opposite was found for the influence of family, which was significantly higher among pregnant women, compared to mothers (36.8%, 10.6%). Furthermore, once a woman had heard about GUMs, 60% believed older infants needed them, and there were no significant differences on this belief between pregnant women and mothers. When asked about the perceived benefits of GUMs, the most frequent responses that women gave were: (i) that it was good for the child's future potential, brain development, and growth (30.8%, 17.2%, and 15.2%, respectively), and (ii) that they perceived that breast‐milk didn't have sufficient nutrients (16.2%). This belief was significantly more common among pregnant women, compared with mothers (28.9% vs. 13.1%).

**Table 3:** Descriptive behaviour characteristics around growing‐on milks (GUM) among Mexican women, stratified by pregnancy/motherhood status

	Total	Pregnant women	Mothers w/children 0–18 m	*p* value
Sample	*n* = 1044	*n* = 296	*n* = 748	
Having heard about GUM, *n* (%)				
Yes	198 (18.97)	38 (12.84)	160 (21.39)	0.003
No	840 (80.46)	256 (86.46)	584 (78.07)	
Don't know	6 (0.57)	2 (0.68)	1 (0.13)	
Intention or actual use of GUM, *n* (%)				
Yes	154 (14.75)	25 (8.45)	129 (17.25)	<0.0001
No	890 (85.25)	271 (91.55)	619 (82.75)	
*Among those who have heard of GUM*				
Sample	*n* = 198	*n* = 38	*n* = 160	
How did you find out about GUM, *n* (%)				
Friends & other mothers	7 (3.54)	1 (2.63)	6 (3.75)	0.99
Family	31 (15.66)	14 (36.84)	17 (10.63)	<0.0001
Health professionals	86 (43.43)	7 (18.42)	79 (49.38)	0.001
Social media, helpline, baby club	1 (0.51)	0 (0.00)	1 (0.63)	0.99
Advertisements	19 (9.60)	2 (5.26)	17 (10.63)	0.53
Other	34 (17.17)	9 (23.68)	25 (15.63)	0.23
Don't know	20 (10.10)	5 (13.16)	15 (9.38)	0.54
Belief baby needs GUM, *n* (%)				
Yes	119 (60.10)	19 (50.00)	100 (62.50)	0.27
No	77 (38.89)	19 (50.00)	58 (36.25)	
Don't know	2 (1.01)	0 (0.00)	2 (1.25)	
Perceived benefits of GUM, *n* (%)				
Perceived baby's behaviour				
Less hungry	29 (14.65)	5 (13.16)	24 (15.00)	0.77
Less crying	1 (0.51)	0 (0.00)	1 (0.63)	0.19
Better sleep	7 (3.54)	0 0.00)	7 (4.38)	0.35
Perceived nutritional benefits				
Iron	17 (8.59)	1 (2.63)	16 (10.00)	0.20
Prebiotics	5 (2.53)	2 (5.26)	3 (1.88)	0.24
DHA	9 (4.55)	0 (0.00)	9 (5.63)	0.21
Perceived benefits for baby's development				
Brain development	34 (17.17)	6 (15.79)	28 (17.50)	0.80
Baby's future	61 (30.81)	11 (28.95)	50 (31.25)	0.78
Baby's growth	38 (15.15)	5 (13.16)	25 (15.63)	0.70
Perceived need due to…				
Allergies	24 (12.12)	6 (15.79)	18 (11.25)	0.41
Breast‐milk not enough benefits	32 (16.16)	11 (28.95)	21 (13.13)	0.01
Cow's milk not enough nutrients	8 (4.04)	2 (5.26)	6 (3.75)	0.65
Other perceived benefits	8 (4.04)	0 (0.00)	8 (5.00)	0.35
Don't know	12 (6.06)	1 (2.63)	11 (6.88)	0.46
*Among those intending or using GUM*				
Sample	*n* = 154	*n* = 25	*n* = 129	
Who recommended using GUM, *n* (%)				
Spouse	5 (3.25)	1 (4.00)	4 (3.10)	0.99
Mother/Mother‐in‐law	34 (22.08)	8 (32.00)	26 (20.16)	0.19
Other family members	21 (13.64)	7 (28.00)	14 (10.85)	0.04
Friends	14 (9.09)	4 (16.00)	10 (7.75)	0.24
Social media	1 (0.65)	0 (0.00)	1 (0.78)	0.99
People in the community	2 (1.30)	1 (4.00)	1 (0.78)	0.29
People I follow on TV/Radio/Social Media	3 (1.95)	0 (0.00)	3 (2.33)	0.99
Science experts	1 (0.65)	0 (0.00)	1 (0.78)	0.99
Doctors	114 (74.03)	13 (52.00)	101 (78.29)	0.01
Nurses/Midwifes	3 (1.95)	0 (0.00)	3 (2.33)	0.99
Pharmacists	3 (1.95)	0 (0.00)	3 (2.33)	0.99

Almost 15% of the participants stated their intention to use or actual use of GUMs, and this proportion was significantly higher among mothers than pregnant women (12.3% vs. 8.5%). For participants who reported intentions to use or actual use of GUMs, the majority reported that the doctor had recommended such product (74%), and this proportion was larger among mothers than pregnant women (78.3% vs. 52%). Mothers and mothers‐in‐law, and other family members were the other actors recommending GUMs (22.1% and 12.6%, respectively) to pregnant women and mothers.

## 4 DISCUSSION

A better understanding of how Mexican mothers and pregnant women were exposed to FUFs and GUMs was facilitated by following the constructs of the TRA. Women were aware of these products since pregnancy, but mothers were even more aware. The study showed that once women were aware of FUFs and GUMs, most of them believed that infants and young children needed these products and that they would benefit from them. Women specifically reported that these products improve brain development and are, therefore, beneficial to the child's future. This is consistent with BMS product claims that imply that the FUF and GUM's ingredients will benefit older infants and young children's brain and cognitive development (Duffy et al., 2021a; Romo‐Palafox et al., 2020), although such claims are often unfounded or poorly substantiated (Munblit, Crawley, et al., 2020). The current regulatory environment in Mexico, including the partial adherence to the Code, is permissive about such claims and influences caregivers' decisions on infant and young child feeding choices, as FUFs are portrayed as equivalent to, or even better than breast‐milk, while GUMs are portrayed as a better option than cow's milk (Romo‐Palafox et al., 2020). This is concerning, especially for GUMs, since these products are not recommended by the WHO and have been declared unnecessary by the WHA. Similarly, GUMs are not recommended by paediatric health organisations, as they are not needed to meet nutritional requirements, and have excessive amounts of added sugars (Duffy et al., 2021b; Romo‐Palafox et al., 2020).

This study also documented that mothers believed that FUFs and GUMs can help manage child allergies. This is consistent with prior studies reporting that specialised BMS products, advertised for managing cow's milk allergy (CMA), have increased in sales without evidence of a corresponding increase in allergies' prevalence (Munblit, Perkin, et al., 2020; van Tulleken, 2018). This is an example of commercially driven overdiagnosis of a condition (van Tulleken, 2018), which is concerning, because CMA overdiagnosis can also undermine mothers' self‐confidence in the quality of their breast‐milk. Since they may fear that if they drink cow's milk, their breast‐milk may result in allergies in the child, it could lead them to make unwarranted changes in the mothers diets (Munblit, Perkin, et al., 2020; van Tulleken, 2018). In addition, overdiagnosis of CMA results in a financial burden to families, due to the high cost of specialized BMS (Munblit, Perkin, et al., 2020).

A central finding of this study is the large influence that health providers, and specifically doctors have on informing about and recommending FUFs and GUMs, which is likely to affect preferences and beliefs among pregnant women and mothers. Health providers were indeed identified as being a major channel through which mothers became aware of FUFs and GUMs. Health providers, particularly doctors, did not only influence awareness of these products among women, but also influenced women's intentions to use or their actual use of these products. This is consistent with prior studies documenting that health providers can serve as marketing channels for formula companies (Rothstein et al., 2020). Some strategies followed by formula companies to incentivize health providers to promote their products are gifts, travel stipends and free BMS samples (Piwoz & Huffman, 2015). In Mexico, as in many other countries, doctors, and to a lesser degree other health providers, are trusted members of society, hence, it should be expected that if they inform about and recommend FUFs and GUMs, women will be more likely to use them. Other studies have reported that when health providers communicate that BMS “help to fill up” or “supplement” the child's nutrition, they undermine mothers' breastfeeding self‐efficacy as, among other things, it exacerbates the perceptions of breast‐milk insufficiency (Hernández‐Cordero et al., 2020; Rothstein et al., 2020) in terms of volume and/or nutritional quality. This is not a trivial outcome, as in low‐resource settings, BMS use can disrupt the household economy impacting negatively on food security and introduce unnecessary health risks to the child through unsafe preparation or “formula stretching” (Rothstein et al., 2021).

This study also identified family and friends as key influencers in exposing and recommending mothers, and especially pregnant women, to FUFs and GUMs. This is consistent with prior studies in Mexico that identified family members' recommendations of BMS as barriers to breastfeeding (Hernández‐Cordero et al., 2020). Such recommendations are also expected to diminish mothers' breastfeeding self‐efficacy and increase the likelihood of perceptions of insufficient milk. In addition, marketing strategies are likely to influence family, friends, as well as mothers and pregnant women. For example, by portraying claims as statements about the overall healthfulness of FUFs and GUMs—which can include aspects such as environmentally friendly packaging and using organic components—these products have a “health halo effect” that can influence consumer's choice (Duffy et al., 2021a).

Direct FUFs and GUMs advertisement exposure through media channels (e.g., TV, radio, social media) was not as prevalent as expected among the women participating in our study. Methodological issues could explain this finding, as no visual recall‐aids were used to facility their memory of exposure, in the context of the thousands of adverts for a wide range of commercial products that they see on a regular basis. In addition, there can be strong recall bias when it comes to digital advertisement. For example, it has been documented that mothers and pregnant women may not recall digital advertisements as an explicit marketing strategies, as digital advertisement results from data‐brokers who apply machine learning techniques to tailor marketing in personal ways (Wilking, 2020).

This study is innovative because, as far as we know, it is the first documenting intention to use and actual use of FUFs and GUMs in Mexico. However, we acknowledge that it has some limitations. First, because of the way the questions were asked, it was not possible to separate intention from actual use of FUFs and GUMs. Second, the study was only conducted in two large cities of the country, hence, the generalisation of the findings should be interpreted with caution. Third, questions about channels of initial exposure to FUFs and GUMs (i.e., how women found out about FUFs and GUMs), did not allow to disaggregate answers by type of providers (i.e., nurses, doctors, pharmacists). Analysis of users and mothers intending to use FUFs and GUMs allowed such disaggreation showing the particular role of doctors in recommending these products. Fourth, the survey was collected among mothers with infants and young children 0–18 months. Nevertheless, GUMs are targeted up to 36 months so the findings are related to the initial months of the use of GUMs. Further studies would benefit to expand this age range to assess if the beliefs, normative referents and use holds throughout the period that GUMs are marketed to. Finally, the analyses only followed a descriptive perspective. Future studies should assess the actual associations between constructs through more robust models, such as structural equation models.

## 5 CONCLUSIONS

BMS marketing, including FUFs and GUMs, represent a major threat to advancing breastfeeding in Mexico. It is important to note, however, that despite the constant marketing influence of BMS in the country, Mexico has managed to show improvements in breastfeeding outcomes over the past decade. For example, in Mexico, exclusive breastfeeding rate among <6 months‐old infants improved from 14.4% in 2012 to 28.3% in 2018 (Gonzalez de Cosio et al., 2013; Rivera‐Dommarco et al., 2020). Although insufficient to meet the World Health Assembly's target of 50% by 2025, these improvements need to be recognised, as they are likely to be linked to sustained political support, advocacy, and research efforts (Unar‐Munguía et al., 2020). In Mexico, actions from intersectoral groups like the Becoming Breastfeeding Friendly (BBF) Committee (Safon et al., 2018), the sustained advocacy efforts of local non‐for profit and international organisations, the commitment of some governmental actors in implementing nationwide programs, and the sustained research and monitoring efforts from local universities and research institutes may have helped counteract some of the BMS industry marketing pressures (Buccini et al., 2020). However, despite such improvements in breastfeeding practices, there is still a low to moderate scaling‐up environment for breastfeeding in Mexico (González de Cosío et al., 2018). A specific example of the institutional fragility is that Mexico has not updated its regulations regarding FUFs and GUMs as urged in the WHA 69.9 resolution of 2016 (Grummer‐Strawn et al., 2017).

There is still a long way to reach global breastfeeding targets and the relentless marketing of FUFs and GUMs needs to be addressed to enhance and sustain these improvements. Allowing unrestricted promotion of FUFs and GUMs to the public and health providers needs to be urgently addressed. This could be achieved by, first, limiting the ways in which BMS manufactures can incentivize health providers, but also by educating them with regard to conflict of interest issues and the risks associated with recommending FUFs and GUMs. It is crucial that health providers, particularly doctors, understand the effects that their advice can have on seriously undermining breastfeeding self‐efficacy and increase the risk of perceptions of insufficient milk among mothers, both in terms of quantity and quality of their milk. A second way is to prohibit nutrition and health claims on FUFs and GUMs through regulation, in line with the Code. No claims should be allowed in line with the Code and Codex standard. There is a need for systematic regulatory oversight and adequate FOP labelling of FUFs and GUMs, especially with respect to added sugars. Perhaps Mexico could take advantage of the recently approved FOP warning labels legislation (Pérez‐Escamilla et al., 2021), that currently excludes BMS. Third, the findings suggest that the Code needs to be adequately enforced. Attempts to promote the Code enforcement have been pushed for decades in Mexico without tangible results. This suggests finding new and innovative approaches to achieve better behaviours, for example, exploring economic behavioural models (Hernández‐Cordero et al., 2019; Lozada‐Tequeanes et al., 2020).

## CONFLICTS OF INTEREST

The authors declare no conflict of interest.

## AUTHOR CONTRIBUTIONS

Mireya Vilar‐Compte: conceptualised the study, contributed in the data analysis, drafted the full manuscript. Sonia Hernández Cordero: contributed in conceptualising the study, contributed in the data analysis, provided a critical review of the full manuscript. Ana C. Castañeda‐Márquez: conducted the data management and analysis. Nigel Rollins: provided feedback on the study design and interpretation. Gillian Kingston: provided feedback on the study design and interpretation. Rafael Pérez‐Escamilla: contributed in conceptualising the study, provided a critical review of the full manuscript.

## Data Availability

The data that support the findings of this study are available from the corresponding author upon reasonable request after receiving collaborative approval from the co‐authors.
